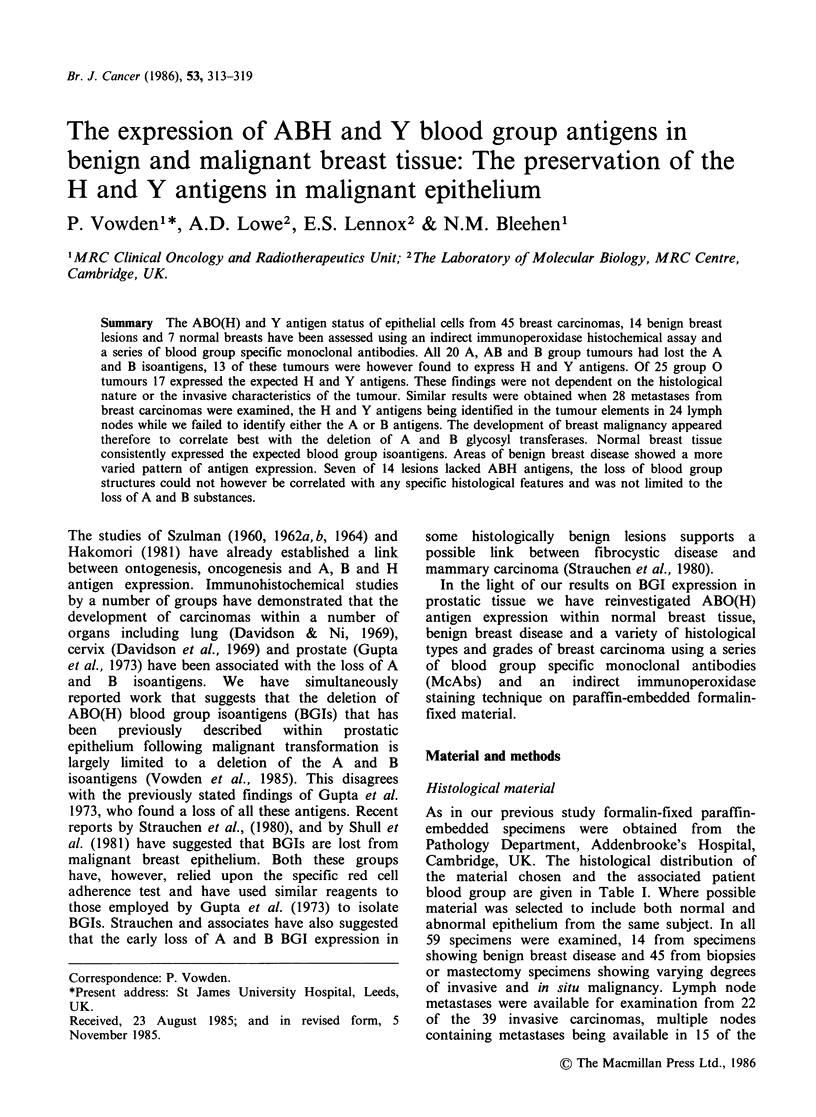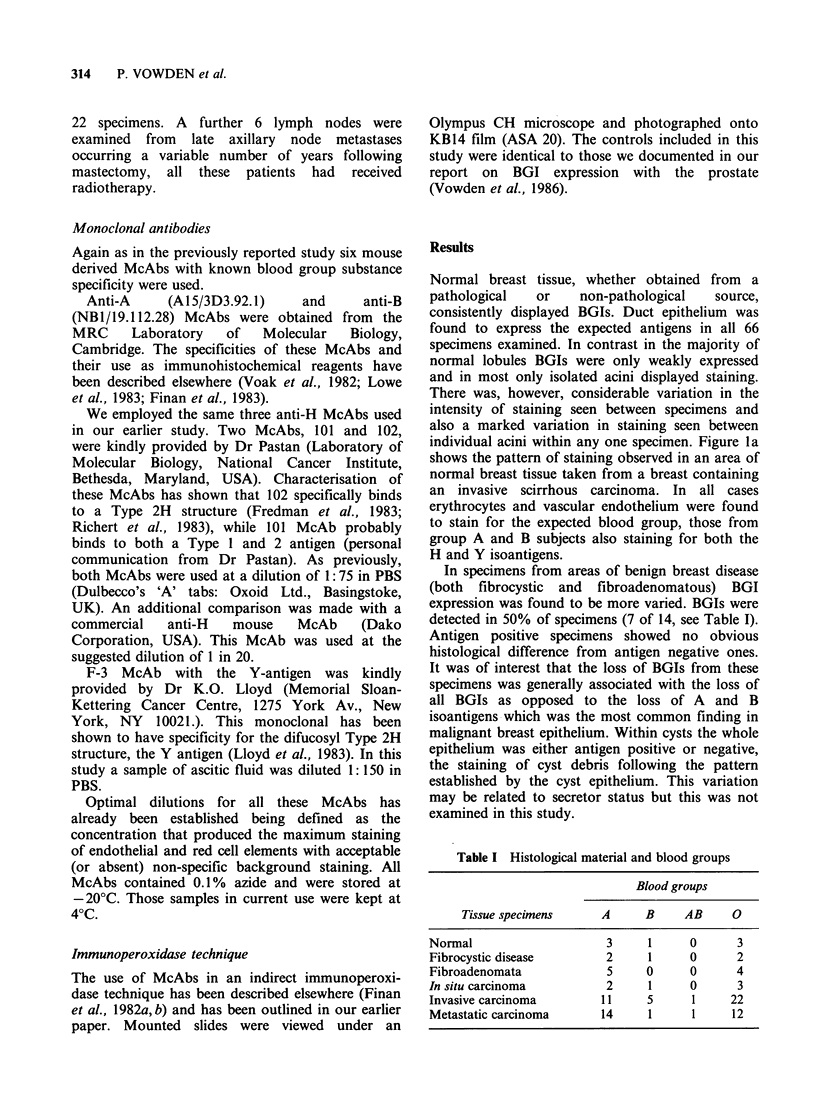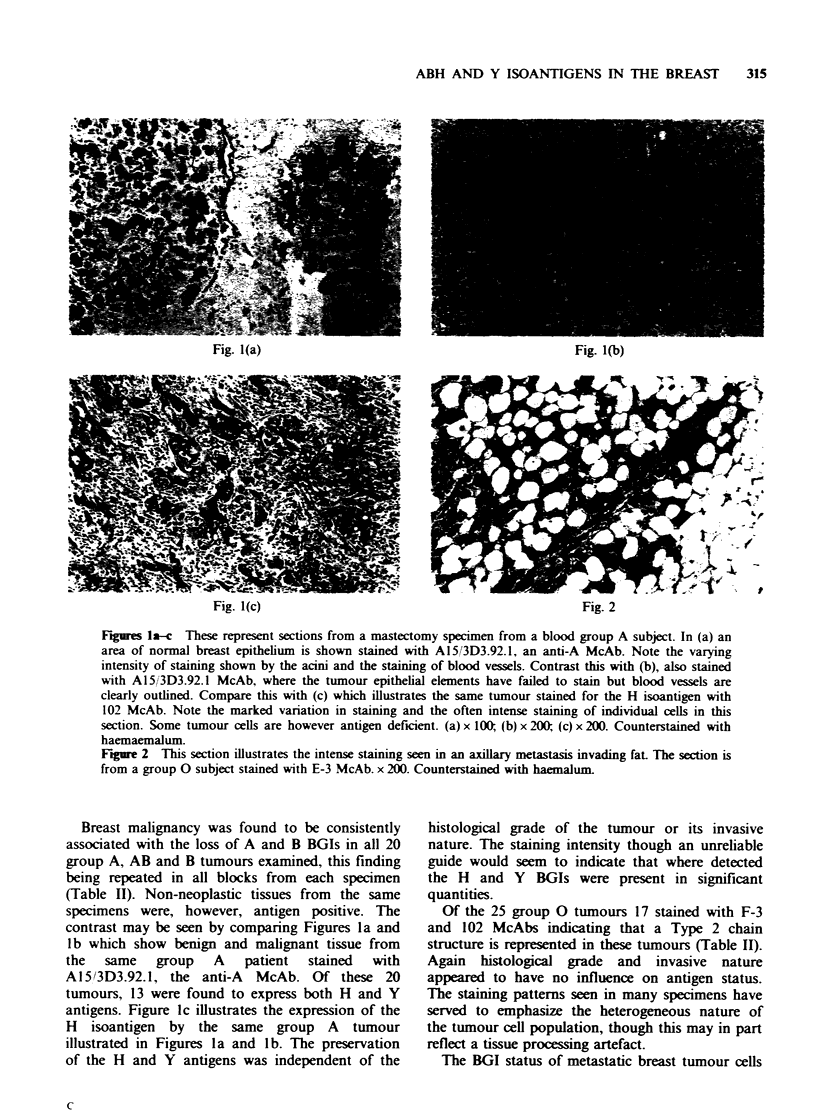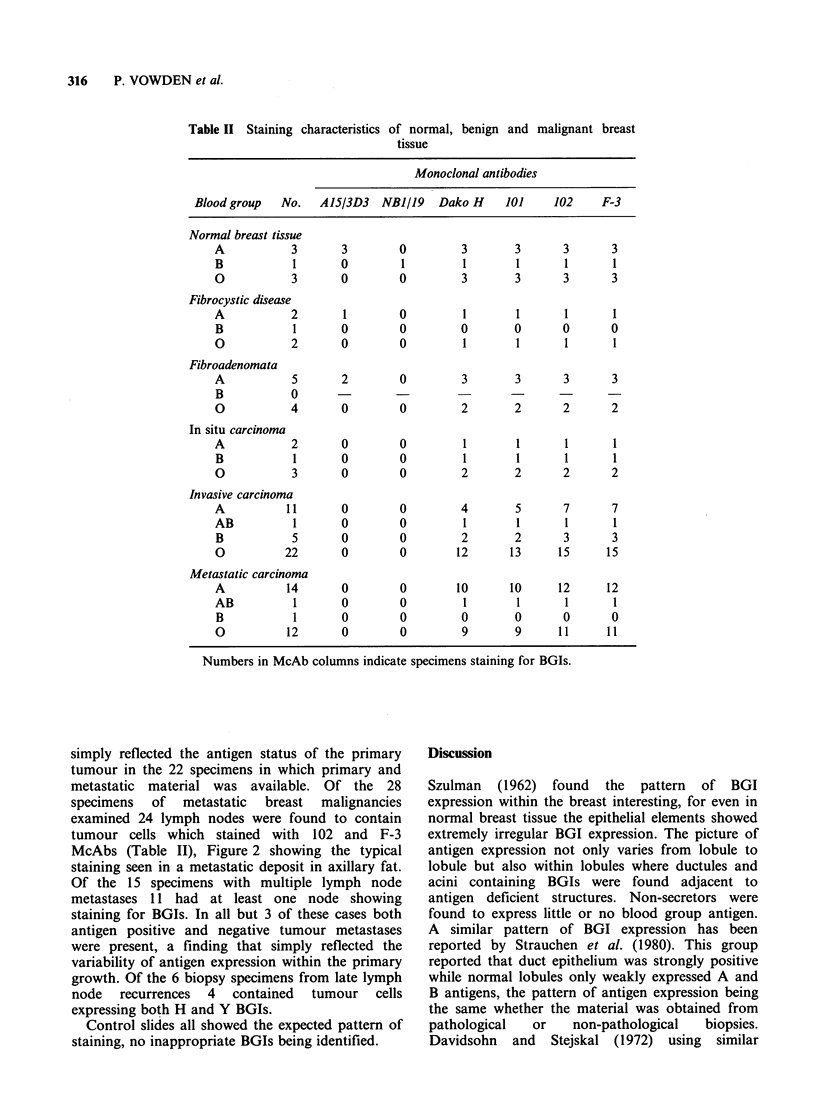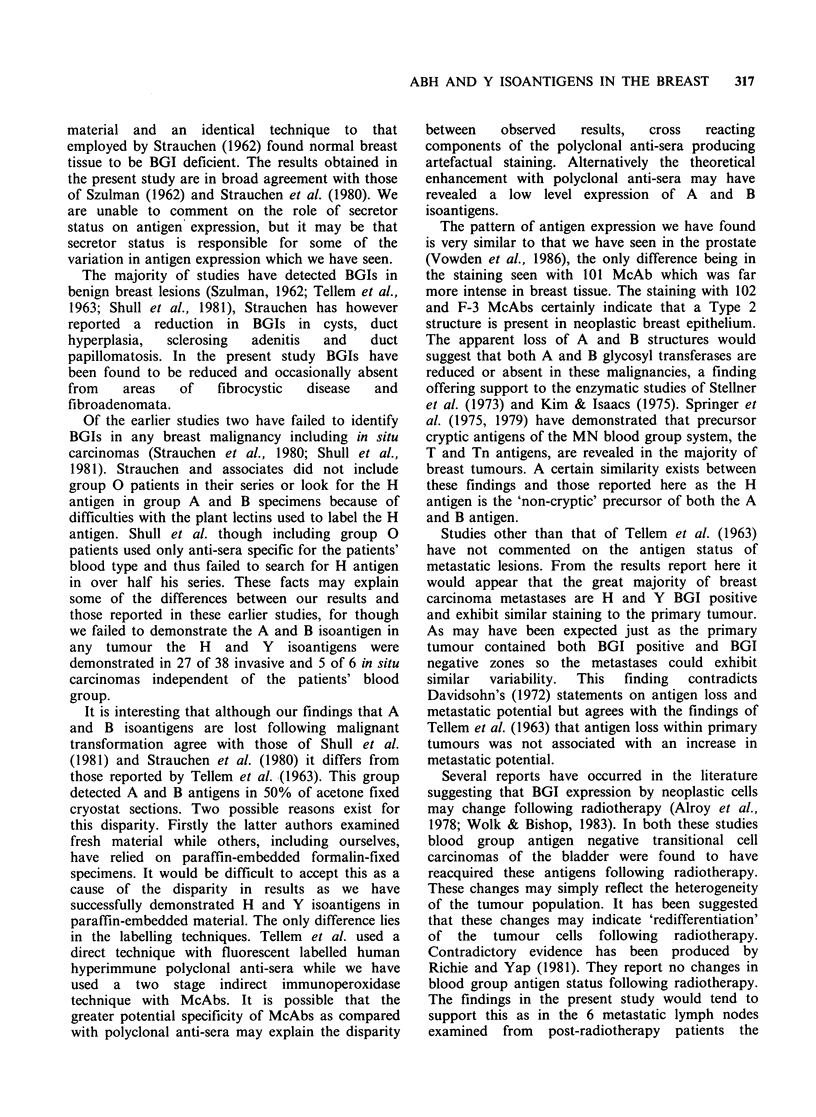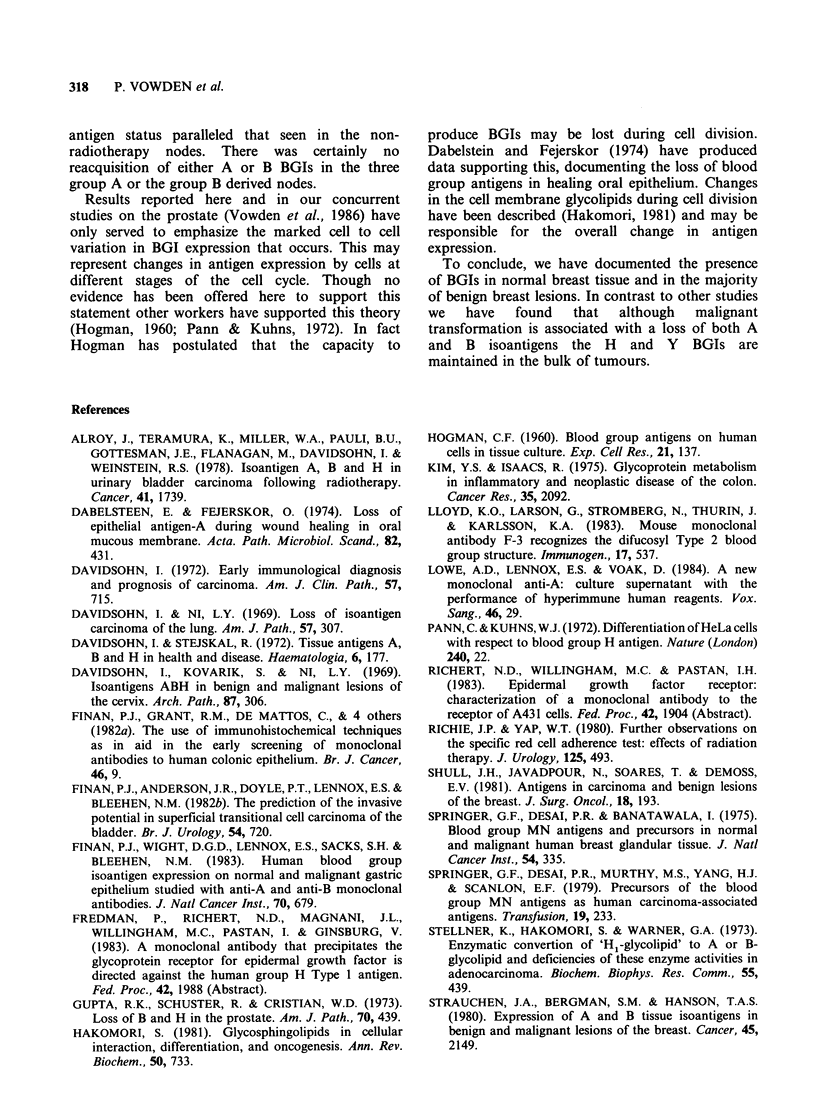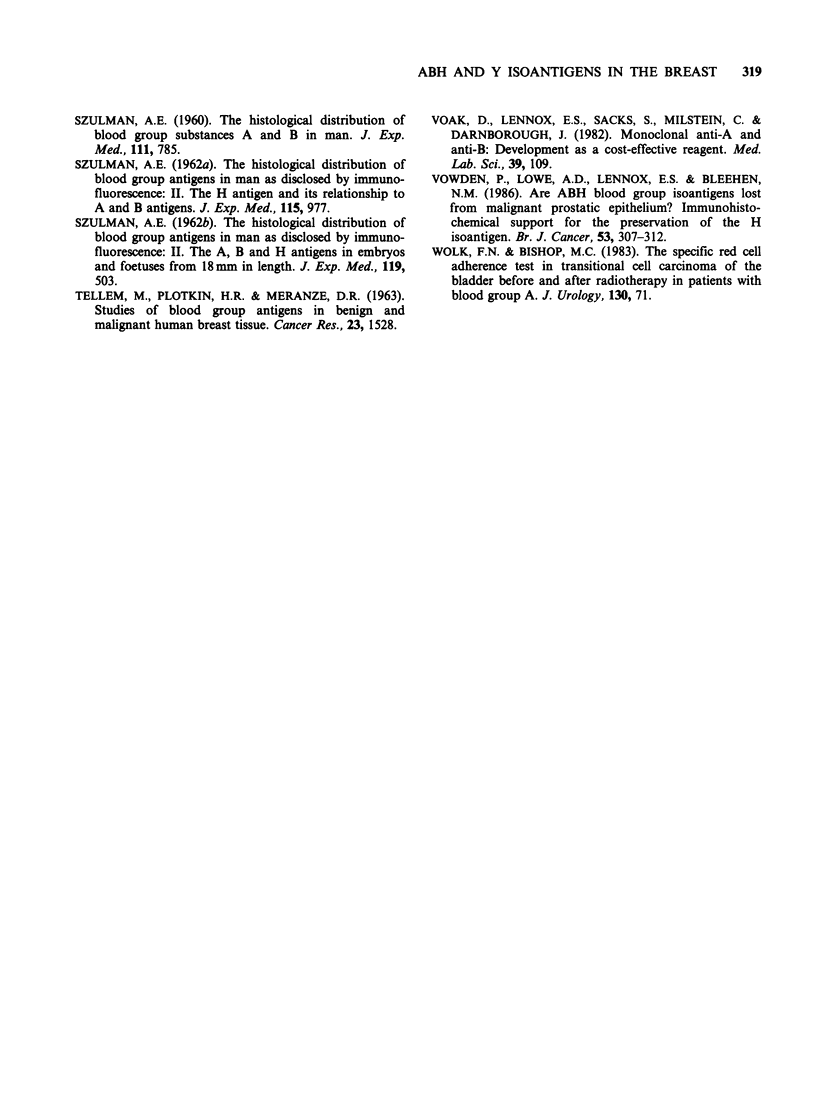# The expression of ABH and Y blood group antigens in benign and malignant breast tissue: the preservation of the H and Y antigens in malignant epithelium.

**DOI:** 10.1038/bjc.1986.54

**Published:** 1986-03

**Authors:** P. Vowden, A. D. Lowe, E. S. Lennox, N. M. Bleehen

## Abstract

**Images:**


*Br. J. Cancer* (1986), **53**, 313–319

### The expression of ABH and Y blood group antigens in benign and malignant breast tissue: The preservation of the H and Y antigens in malignant epithelium

P. Vowden[1]*, A.D. Lowe[2], E.S. Lennox[2] & N.M. Bleehen[1]

[1]*MRC Clinical Oncology and Radiotherapeutics Unit;* [2]*The Laboratory of Molecular Biology, MRC Centre, Cambridge, UK.*

**Summary** The ABO(H) and Y antigen status of epithelial cells from 45 breast carcinomas, 14 benign breast lesions and 7 normal breasts have been assessed using an indirect immunoperoxidase histochemical assay and a series of blood group specific monoclonal antibodies. All 20 A, AB and B group tumours had lost the A and B isoantigens, 13 of these tumours were however found to express H and Y antigens. Of 25 group O tumours 17 expressed the expected H and Y antigens. These findings were not dependent on the histological nature or the invasive characteristics of the tumour. Similar results were obtained when 28 metastases from breast carcinomas were examined, the H and Y antigens being identified in the tumour elements in 24 lymph nodes while we failed to identify either the A or B antigens. The development of breast malignancy appeared therefore to correlate best with the deletion of A and B glycosyl transferases. Normal breast tissue consistently expressed the expected blood group isoantigens. Areas of benign breast disease showed a more varied pattern of antigen expression. Seven of 14 lesions lacked ABH antigens, the loss of blood group structures could not however be correlated with any specific histological features and was not limited to the loss of A and B substances.

The studies of Szulman (1960, 1962*a*, *b*, 1964) and Hakomori (1981) have already established a link between ontogenesis, oncogenesis and A, B and H antigen expression. Immunohistochemical studies by a number of groups have demonstrated that the development of carcinomas within a number of organs including lung (Davidson & Ni, 1969), cervix (Davidson *et al.*, 1969) and prostate (Gupta *et al.*, 1973) have been associated with the loss of A and B isoantigens. We have simultaneously reported work that suggests that the deletion of ABO(H) blood group isoantigens (BGIs) that has been previously described within prostatic epithelium following malignant transformation is largely limited to a deletion of the A and B isoantigens (Vowden *et al.*, 1985). This disagrees with the previously stated findings of Gupta *et al.* 1973, who found a loss of all these antigens. Recent reports by Strauchen *et al.*, (1980), and by Shull *et al.* (1981) have suggested that BGIs are lost from malignant breast epithelium. Both these groups have, however, relied upon the specific red cell adherence test and have used similar reagents to those employed by Gupta *et al.* (1973) to isolate BGIs. Strauchen and associates have also suggested that the early loss of A and B BGI expression in some histologically benign lesions supports a possible link between fibrocystic disease and mammary carcinoma (Strauchen *et al.*, 1980).

In the light of our results on BGI expression in prostatic tissue we have reinvestigated ABO(H) antigen expression within normal breast tissue, benign breast disease and a variety of histological types and grades of breast carcinoma using a series of blood group specific monoclonal antibodies (McAbs) and an indirect immunoperoxidase staining technique on paraffin-embedded formalin-fixed material.

### Material and methods

### *Histological material*

As in our previous study formalin-fixed paraffin-embedded specimens were obtained from the Pathology Department, Addenbrooke's Hospital, Cambridge, UK. The histological distribution of the material chosen and the associated patient blood group are given in Table I. Where possible material was selected to include both normal and abnormal epithelium from the same subject. In all 59 specimens were examined, 14 from specimens showing benign breast disease and 45 from biopsies or mastectomy specimens showing varying degrees of invasive and *in situ* malignancy. Lymph node metastases were available for examination from 22 of the 39 invasive carcinomas, multiple nodes containing metastases being available in 15 of the

Correspondence: P. Vowden.
*Present address: St James University Hospital, Leeds, UK.
Received, 23 August 1985; and in revised form, 5 November 1985.

© The Macmillan Press Ltd., 1986

22 specimens. A further 6 lymph nodes were examined from late axillary node metastases occurring a variable number of years following mastectomy, all these patients had received radiotherapy.

*Monoclonal antibodies*

Again as in the previously reported study six mouse derived McAbs with known blood group substance specificity were used.

Anti-A (A15/3D3.92.1) and anti-B (NB1/19.112.28) McAbs were obtained from the MRC Laboratory of Molecular Biology, Cambridge. The specificities of these McAbs and their use as immunohistochemical reagents have been described elsewhere (Voak *et al.*, 1982; Lowe *et al.*, 1983; Finan *et al.*, 1983).

We employed the same three anti-H McAbs used in our earlier study. Two McAbs, 101 and 102, were kindly provided by Dr Pastan (Laboratory of Molecular Biology, National Cancer Institute, Bethesda, Maryland, USA). Characterisation of these McAbs has shown that 102 specifically binds to a Type 2H structure (Fredman *et al.*, 1983; Richert *et al.*, 1983), while 101 McAb probably binds to both a Type 1 and 2 antigen (personal communication from Dr Pastan). As previously, both McAbs were used at a dilution of 1:75 in PBS (Dulbecco's 'A' tabs: Oxoid Ltd., Basingstoke, UK). An additional comparison was made with a commercial anti-H mouse McAb (Dako Corporation, USA). This McAb was used at the suggested dilution of 1 in 20.

F-3 McAb with the Y-antigen was kindly provided by Dr K.O. Lloyd (Memorial Sloan-Kettering Cancer Centre, 1275 York Av., New York, NY 10021.). This monoclonal has been shown to have specificity for the difucosyl Type 2H structure, the Y antigen (Lloyd *et al.*, 1983). In this study a sample of ascitic fluid was diluted 1:150 in PBS.

Optimal dilutions for all these McAbs has already been established being defined as the concentration that produced the maximum staining of endothelial and red cell elements with acceptable (or absent) non-specific background staining. All McAbs contained 0.1% azide and were stored at −20°C. Those samples in current use were kept at 4°C.

*Immunoperoxidase technique*

The use of McAbs in an indirect immunoperoxidase technique has been described elsewhere (Finan *et al.*, 1982*a*, *b*) and has been outlined in our earlier paper. Mounted slides were viewed under an Olympus CH microscope and photographed onto KB14 film (ASA 20). The controls included in this study were identical to those we documented in our report on BGI expression with the prostate (Vowden *et al.*, 1986).

### Results

Normal breast tissue, whether obtained from a pathological or non-pathological source, consistently displayed BGIs. Duct epithelium was found to express the expected antigens in all 66 specimens examined. In contrast in the majority of normal lobules BGIs were only weakly expressed and in most only isolated acini displayed staining. There was, however, considerable variation in the intensity of staining seen between specimens and also a marked variation in staining seen between individual acini within any one specimen. Figure 1a shows the pattern of staining observed in an area of normal breast tissue taken from a breast containing an invasive scirrhous carcinoma. In all cases erythrocytes and vascular endothelium were found to stain for the expected blood group, those from group A and B subjects also staining for both the H and Y isoantigens.

In specimens from areas of benign breast disease (both fibrocystic and fibroadenomatous) BGI expression was found to be more varied. BGIs were detected in 50% of specimens (7 of 14, see Table I). Antigen positive specimens showed no obvious histological difference from antigen negative ones. It was of interest that the loss of BGIs from these specimens was generally associated with the loss of all BGIs as opposed to the loss of A and B isoantigens which was the most common finding in malignant breast epithelium. Within cysts the whole epithelium was either antigen positive or negative, the staining of cyst debris following the pattern established by the cyst epithelium. This variation may be related to secretor status but this was not examined in this study.

**Table I** Histological material and blood groups

| | *Blood groups* | | | |
|---|---|---|---|---|
| *Tissue specimens* | *A* | *B* | *AB* | *O* |
| Normal | 3 | 1 | 0 | 3 |
| Fibrocystic disease | 2 | 1 | 0 | 2 |
| Fibroadenomata | 5 | 0 | 0 | 4 |
| *In situ* carcinoma | 2 | 1 | 0 | 3 |
| Invasive carcinoma | 11 | 5 | 1 | 22 |
| Metastatic carcinoma | 14 | 1 | 1 | 12 |

Fig. 1(a)

Fig. 1(b)

Fig. 1(c)

Fig. 2

**Figures 1a–c** These represent sections from a mastectomy specimen from a blood group A subject. In (a) an area of normal breast epithelium is shown stained with A15/3D3.92.1, an anti-A McAb. Note the varying intensity of staining shown by the acini and the staining of blood vessels. Contrast this with (b), also stained with A15/3D3.92.1 McAb, where the tumour epithelial elements have failed to stain but blood vessels are clearly outlined. Compare this with (c) which illustrates the same tumour stained for the H isoantigen with 102 McAb. Note the marked variation in staining and the often intense staining of individual cells in this section. Some tumour cells are however antigen deficient. (a) × 100; (b) × 200; (c) × 200. Counterstained with haemaemalum.

**Figure 2** This section illustrates the intense staining seen in an axillary metastasis invading fat. The section is from a group O subject stained with E-3 McAb. × 200. Counterstained with haemalum.

Breast malignancy was found to be consistently associated with the loss of A and B BGIs in all 20 group A, AB and B tumours examined, this finding being repeated in all blocks from each specimen (Table II). Non-neoplastic tissues from the same specimens were, however, antigen positive. The contrast may be seen by comparing Figures 1a and 1b which show benign and malignant tissue from the same group A patient stained with A15/3D3.92.1, the anti-A McAb. Of these 20 tumours, 13 were found to express both H and Y antigens. Figure 1c illustrates the expression of the H isoantigen by the same group A tumour illustrated in Figures 1a and 1b. The preservation of the H and Y antigens was independent of the histological grade of the tumour or its invasive nature. The staining intensity though an unreliable guide would seem to indicate that where detected the H and Y BGIs were present in significant quantities.

Of the 25 group O tumours 17 stained with F-3 and 102 McAbs indicating that a Type 2 chain structure is represented in these tumours (Table II). Again histological grade and invasive nature appeared to have no influence on antigen status. The staining patterns seen in many specimens have served to emphasize the heterogeneous nature of the tumour cell population, though this may in part reflect a tissue processing artefact.

The BGI status of metastatic breast tumour cells

**Table II** Staining characteristics of normal, benign and malignant breast tissue

| | | Monoclonal antibodies | | | | | |
|---|---|---|---|---|---|---|---|
| *Blood group* | *No.* | *A15/3D3* | *NB1/19* | *Dako H* | *101* | *102* | *F-3* |
| *Normal breast tissue* | | | | | | | |
| A | 3 | 3 | 0 | 3 | 3 | 3 | 3 |
| B | 1 | 0 | 1 | 1 | 1 | 1 | 1 |
| O | 3 | 0 | 0 | 3 | 3 | 3 | 3 |
| *Fibrocystic disease* | | | | | | | |
| A | 2 | 1 | 0 | 1 | 1 | 1 | 1 |
| B | 1 | 0 | 0 | 0 | 0 | 0 | 0 |
| O | 2 | 0 | 0 | 1 | 1 | 1 | 1 |
| *Fibroadenomata* | | | | | | | |
| A | 5 | 2 | 0 | 3 | 3 | 3 | 3 |
| B | 0 | — | — | — | — | — | — |
| O | 4 | 0 | 0 | 2 | 2 | 2 | 2 |
| In situ *carcinoma* | | | | | | | |
| A | 2 | 0 | 0 | 1 | 1 | 1 | 1 |
| B | 1 | 0 | 0 | 1 | 1 | 1 | 1 |
| O | 3 | 0 | 0 | 2 | 2 | 2 | 2 |
| *Invasive carcinoma* | | | | | | | |
| A | 11 | 0 | 0 | 4 | 5 | 7 | 7 |
| AB | 1 | 0 | 0 | 1 | 1 | 1 | 1 |
| B | 5 | 0 | 0 | 2 | 2 | 3 | 3 |
| O | 22 | 0 | 0 | 12 | 13 | 15 | 15 |
| *Metastatic carcinoma* | | | | | | | |
| A | 14 | 0 | 0 | 10 | 10 | 12 | 12 |
| AB | 1 | 0 | 0 | 1 | 1 | 1 | 1 |
| B | 1 | 0 | 0 | 0 | 0 | 0 | 0 |
| O | 12 | 0 | 0 | 9 | 9 | 11 | 11 |

Numbers in McAb columns indicate specimens staining for BGIs.

simply reflected the antigen status of the primary tumour in the 22 specimens in which primary and metastatic material was available. Of the 28 specimens of metastatic breast malignancies examined 24 lymph nodes were found to contain tumour cells which stained with 102 and F-3 McAbs (Table II), Figure 2 showing the typical staining seen in a metastatic deposit in axillary fat. Of the 15 specimens with multiple lymph node metastases 11 had at least one node showing staining for BGIs. In all but 3 of these cases both antigen positive and negative tumour metastases were present, a finding that simply reflected the variability of antigen expression within the primary growth. Of the 6 biopsy specimens from late lymph node recurrences 4 contained tumour cells expressing both H and Y BGIs.

Control slides all showed the expected pattern of staining, no inappropriate BGIs being identified.

### Discussion

Szulman (1962) found the pattern of BGI expression within the breast interesting, for even in normal breast tissue the epithelial elements showed extremely irregular BGI expression. The picture of antigen expression not only varies from lobule to lobule but also within lobules where ductules and acini containing BGIs were found adjacent to antigen deficient structures. Non-secretors were found to express little or no blood group antigen. A similar pattern of BGI expression has been reported by Strauchen *et al.* (1980). This group reported that duct epithelium was strongly positive while normal lobules only weakly expressed A and B antigens, the pattern of antigen expression being the same whether the material was obtained from pathological or non-pathological biopsies. Davidsohn and Stejskal (1972) using similar material and an identical technique to that employed by Strauchen (1962) found normal breast tissue to be BGI deficient. The results obtained in the present study are in broad agreement with those of Szulman (1962) and Strauchen *et al.* (1980). We are unable to comment on the role of secretor status on antigen expression, but it may be that secretor status is responsible for some of the variation in antigen expression which we have seen.

The majority of studies have detected BGIs in benign breast lesions (Szulman, 1962; Tellem *et al.*, 1963; Shull *et al.*, 1981), Strauchen has however reported a reduction in BGIs in cysts, duct hyperplasia, sclerosing adenitis and duct papillomatosis. In the present study BGIs have been found to be reduced and occasionally absent from areas of fibrocystic disease and fibroadenomata.

Of the earlier studies two have failed to identify BGIs in any breast malignancy including *in situ* carcinomas (Strauchen *et al.*, 1980; Shull *et al.*, 1981). Strauchen and associates did not include group O patients in their series or look for the H antigen in group A and B specimens because of difficulties with the plant lectins used to label the H antigen. Shull *et al.* though including group O patients used only anti-sera specific for the patients' blood type and thus failed to search for H antigen in over half his series. These facts may explain some of the differences between our results and those reported in these earlier studies, for though we failed to demonstrate the A and B isoantigen in any tumour the H and Y isoantigens were demonstrated in 27 of 38 invasive and 5 of 6 *in situ* carcinomas independent of the patients' blood group.

It is interesting that although our findings that A and B isoantigens are lost following malignant transformation agree with those of Shull *et al.* (1981) and Strauchen *et al.* (1980) it differs from those reported by Tellem *et al.* (1963). This group detected A and B antigens in 50% of acetone fixed cryostat sections. Two possible reasons exist for this disparity. Firstly the latter authors examined fresh material while others, including ourselves, have relied on paraffin-embedded formalin-fixed specimens. It would be difficult to accept this as a cause of the disparity in results as we have successfully demonstrated H and Y isoantigens in paraffin-embedded material. The only difference lies in the labelling techniques. Tellem *et al.* used a direct technique with fluorescent labelled human hyperimmune polyclonal anti-sera while we have used a two stage indirect immunoperoxidase technique with McAbs. It is possible that the greater potential specificity of McAbs as compared with polyclonal anti-sera may explain the disparity between observed results, cross reacting components of the polyclonal anti-sera producing artefactual staining. Alternatively the theoretical enhancement with polyclonal anti-sera may have revealed a low level expression of A and B isoantigens.

The pattern of antigen expression we have found is very similar to that we have seen in the prostate (Vowden *et al.*, 1986), the only difference being in the staining seen with 101 McAb which was far more intense in breast tissue. The staining with 102 and F-3 McAbs certainly indicate that a Type 2 structure is present in neoplastic breast epithelium. The apparent loss of A and B structures would suggest that both A and B glycosyl transferases are reduced or absent in these malignancies, a finding offering support to the enzymatic studies of Stellner *et al.* (1973) and Kim & Isaacs (1975). Springer *et al.* (1975, 1979) have demonstrated that precursor cryptic antigens of the MN blood group system, the T and Tn antigens, are revealed in the majority of breast tumours. A certain similarity exists between these findings and those reported here as the H antigen is the 'non-cryptic' precursor of both the A and B antigen.

Studies other than that of Tellem *et al.* (1963) have not commented on the antigen status of metastatic lesions. From the results report here it would appear that the great majority of breast carcinoma metastases are H and Y BGI positive and exhibit similar staining to the primary tumour. As may have been expected just as the primary tumour contained both BGI positive and BGI negative zones so the metastases could exhibit similar variability. This finding contradicts Davidsohn's (1972) statements on antigen loss and metastatic potential but agrees with the findings of Tellem *et al.* (1963) that antigen loss within primary tumours was not associated with an increase in metastatic potential.

Several reports have occurred in the literature suggesting that BGI expression by neoplastic cells may change following radiotherapy (Alroy *et al.*, 1978; Wolk & Bishop, 1983). In both these studies blood group antigen negative transitional cell carcinomas of the bladder were found to have reacquired these antigens following radiotherapy. These changes may simply reflect the heterogeneity of the tumour population. It has been suggested that these changes may indicate 'redifferentiation' of the tumour cells following radiotherapy. Contradictory evidence has been produced by Richie and Yap (1981). They report no changes in blood group antigen status following radiotherapy. The findings in the present study would tend to support this as in the 6 metastatic lymph nodes examined from post-radiotherapy patients the antigen status paralleled that seen in the non-radiotherapy nodes. There was certainly no reacquisition of either A or B BGIs in the three group A or the group B derived nodes.

Results reported here and in our concurrent studies on the prostate (Vowden et al., 1986) have only served to emphasize the marked cell to cell variation in BGI expression that occurs. This may represent changes in antigen expression by cells at different stages of the cell cycle. Though no evidence has been offered here to support this statement other workers have supported this theory (Hogman, 1960; Pann & Kuhns, 1972). In fact Hogman has postulated that the capacity to produce BGIs may be lost during cell division. Dabelstein and Fejerskor (1974) have produced data supporting this, documenting the loss of blood group antigens in healing oral epithelium. Changes in the cell membrane glycolipids during cell division have been described (Hakomori, 1981) and may be responsible for the overall change in antigen expression.

To conclude, we have documented the presence of BGIs in normal breast tissue and in the majority of benign breast lesions. In contrast to other studies we have found that although malignant transformation is associated with a loss of both A and B isoantigens the H and Y BGIs are maintained in the bulk of tumours.

### References

ALROY, J., TERAMURA, K., MILLER, W.A., PAULI, B.U., GOTTESMAN, J.E., FLANAGAN, M., DAVIDSOHN, I. & WEINSTEIN, R.S. (1978). Isoantigen A, B and H in urinary bladder carcinoma following radiotherapy. *Cancer*, **41**, 1739.

DABELSTEEN, E. & FEJERSKOR, O. (1974). Loss of epithelial antigen-A during wound healing in oral mucous membrane. *Acta. Path. Microbiol. Scand.*, **82**, 431.

DAVIDSOHN, I. (1972). Early immunological diagnosis and prognosis of carcinoma. *Am. J. Clin. Path.*, **57**, 715.

DAVIDSOHN, I. & NI, L.Y. (1969). Loss of isoantigen carcinoma of the lung. *Am. J. Path.*, **57**, 307.

DAVIDSOHN, I. & STEJSKAL, R. (1972). Tissue antigens A, B and H in health and disease. *Haematologia*, **6**, 177.

DAVIDSOHN, I., KOVARIK, S. & NI, L.Y. (1969). Isoantigens ABH in benign and malignant lesions of the cervix. *Arch. Path.*, **87**, 306.

FINAN, P.J., GRANT, R.M., DE MATTOS, C., & 4 others (1982*a*). The use of immunohistochemical techniques as in aid in the early screening of monoclonal antibodies to human colonic epithelium. *Br. J. Cancer*, **46**, 9.

FINAN, P.J., ANDERSON, J.R., DOYLE, P.T., LENNOX, E.S. & BLEEHEN, N.M. (1982*b*). The prediction of the invasive potential in superficial transitional cell carcinoma of the bladder. *Br. J. Urology*, **54**, 720.

FINAN, P.J., WIGHT, D.G.D., LENNOX, E.S., SACKS, S.H. & BLEEHEN, N.M. (1983). Human blood group isoantigen expression on normal and malignant gastric epithelium studied with anti-A and anti-B monoclonal antibodies. *J. Natl Cancer Inst.*, **70**, 679.

FREDMAN, P., RICHERT, N.D., MAGNANI, J.L., WILLINGHAM, M.C., PASTAN, I. & GINSBURG, V. (1983). A monoclonal antibody that precipitates the glycoprotein receptor for epidermal growth factor is directed against the human group H Type 1 antigen. *Fed. Proc.*, **42**, 1988 (Abstract).

GUPTA, R.K., SCHUSTER, R. & CRISTIAN, W.D. (1973). Loss of B and H in the prostate. *Am. J. Path.*, **70**, 439.

HAKOMORI, S. (1981). Glycosphingolipids in cellular interaction, differentiation, and oncogenesis. *Ann. Rev. Biochem.*, **50**, 733.

HOGMAN, C.F. (1960). Blood group antigens on human cells in tissue culture. *Exp. Cell Res.*, **21**, 137.

KIM, Y.S. & ISAACS, R. (1975). Glycoprotein metabolism in inflammatory and neoplastic disease of the colon. *Cancer Res.*, **35**, 2092.

LLOYD, K.O., LARSON, G., STROMBERG, N., THURIN, J. & KARLSSON, K.A. (1983). Mouse monoclonal antibody F-3 recognizes the difucosyl Type 2 blood group structure. *Immunogen.*, **17**, 537.

LOWE, A.D., LENNOX, E.S. & VOAK, D. (1984). A new monoclonal anti-A: culture supernatant with the performance of hyperimmune human reagents. *Vox. Sang.*, **46**, 29.

PANN, C. & KUHNS, W.J. (1972). Differentiation of HeLa cells with respect to blood group H antigen. *Nature* (*London*) **240**, 22.

RICHERT, N.D., WILLINGHAM, M.C. & PASTAN, I.H. (1983). Epidermal growth factor receptor: characterization of a monoclonal antibody to the receptor of A431 cells. *Fed. Proc.*, **42**, 1904 (Abstract).

RICHIE, J.P. & YAP, W.T. (1980). Further observations on the specific red cell adherence test: effects of radiation therapy. *J. Urology*, **125**, 493.

SHULL, J.H., JAVADPOUR, N., SOARES, T. & DEMOSS, E.V. (1981). Antigens in carcinoma and benign lesions of the breast. *J. Surg. Oncol.*, **18**, 193.

SPRINGER, G.F., DESAI, P.R. & BANATAWALA, I. (1975). Blood group MN antigens and precursors in normal and malignant human breast glandular tissue. *J. Natl Cancer Inst.*, **54**, 335.

SPRINGER, G.F., DESAI, P.R., MURTHY, M.S., YANG, H.J. & SCANLON, E.F. (1979). Precursors of the blood group MN antigens as human carcinoma-associated antigens. *Transfusion*, **19**, 233.

STELLNER, K., HAKOMORI, S. & WARNER, G.A. (1973). Enzymatic convertion of '$H_1$-glycolipid' to A or B-glycolipid and deficiencies of these enzyme activities in adenocarcinoma. *Biochem. Biophys. Res. Comm.*, **55**, 439.

STRAUCHEN, J.A., BERGMAN, S.M. & HANSON, T.A.S. (1980). Expression of A and B tissue isoantigens in benign and malignant lesions of the breast. *Cancer*, **45**, 2149.

SZULMAN, A.E. (1960). The histological distribution of blood group substances A and B in man. *J. Exp. Med.*, **111**, 785.

SZULMAN, A.E. (1962*a*). The histological distribution of blood group antigens in man as disclosed by immunofluorescence: II. The H antigen and its relationship to A and B antigens. *J. Exp. Med.*, **115**, 977.

SZULMAN, A.E. (1962*b*). The histological distribution of blood group antigens in man as disclosed by immunofluorescence: II. The A, B and H antigens in embryos and foetuses from 18 mm in length. *J. Exp. Med.*, **119**, 503.

TELLEM, M., PLOTKIN, H.R. & MERANZE, D.R. (1963). Studies of blood group antigens in benign and malignant human breast tissue. *Cancer Res.*, **23**, 1528.

VOAK, D., LENNOX, E.S., SACKS, S., MILSTEIN, C. & DARNBOROUGH, J. (1982). Monoclonal anti-A and anti-B: Development as a cost-effective reagent. *Med. Lab. Sci.*, **39**, 109.

VOWDEN, P., LOWE, A.D., LENNOX, E.S. & BLEEHEN, N.M. (1986). Are ABH blood group isoantigens lost from malignant prostatic epithelium? Immunohistochemical support for the preservation of the H isoantigen. *Br. J. Cancer*, **53**, 307–312.

WOLK, F.N. & BISHOP, M.C. (1983). The specific red cell adherence test in transitional cell carcinoma of the bladder before and after radiotherapy in patients with blood group A. *J. Urology*, **130**, 71.